# Kidney cancer mortality in Spain: geographic patterns and possible hypotheses

**DOI:** 10.1186/1471-2407-8-293

**Published:** 2008-10-09

**Authors:** Gonzalo López-Abente, Nuria Aragonés, Beatriz Pérez-Gómez, Rebeca Ramis, Enrique Vidal, Javier García-Pérez, Pablo Fernández-Navarro, Marina Pollán

**Affiliations:** 1 Cancer and Environmental Epidemiology Area, National Centre for Epidemiology, Carlos III Institute of Health, Sinesio Delgado 6, 28029 Madrid, Spain; 2 CIBER Epidemiología y Salud Pública (CIBERESP), Madrid, Spain

## Abstract

**Background:**

Since the second half of the 1990s, kidney cancer mortality has tended to stabilize and decline in many European countries, due to the decrease in the prevalence of smokers. Nevertheless, incidence of kidney cancer is rising across the sexes in some of these countries, a trend which may possibly reflect the fact that improvements in diagnostic techniques are being outweighed by the increased prevalence of some of this tumor's risk factors. This study sought to: examine the geographic pattern of kidney cancer mortality in Spain; suggest possible hypotheses that would help explain these patterns; and enhance existing knowledge about the large proportion of kidney tumors whose cause remains unknown.

**Methods:**

Smoothed municipal relative risks (RRs) for kidney cancer mortality were calculated in men and women, using the conditional autoregressive model proposed by Besag, York and Molliè. Maps were plotted depicting smoothed relative risk estimates, and the distribution of the posterior probability of RR>1 by sex.

**Results:**

Municipal maps displayed a marked geographic pattern, with excess mortality in both sexes, mainly in towns along the Bay of Biscay, including areas of Asturias, the Basque Country and, to a lesser extent, Cantabria. Among women, the geographic pattern was strikingly singular, not in evidence for any other tumors, and marked by excess risk in towns situated in the Salamanca area and Extremaduran Autonomous Region. This difference would lead one to postulate the existence of different exposures of environmental origin in the various regions.

**Conclusion:**

The reasons for this pattern of distribution are not clear, and it would thus be of interest if the effect of industrial emissions on this disease could be studied. The excess mortality observed among women in towns situated in areas with a high degree of natural radiation could reflect the influence of exposures which derive from the geologic composition of the terrain and then become manifest through the agency of drinking water.

## Background

During the 1980s and 90s, kidney cancer mortality increased throughout Europe. There was a trend towards stabilization in subsequent years, though this was principally in western European countries, with the rates continuing to be very high in the eastern European countries [1]. The incidence of these tumors follows a trend which, though very similar to that of mortality in a good number of countries, is not in others, where it continues to rise across the sexes (e.g., Norway, Ireland, UK England, UK Scotland) [1,2]. In the closing decades of the 20^th ^century, mortality due to this tumor registered an annual increase in Spain of 2.9% among men and 1.4% among women [3], and it is estimated that there were approximately 4000 new cases in 2002 [4]. The most frequent histologic type of kidney cancer in Spain is renal cell carcinoma (RCC) (accounting for around 80% of cases), followed by transitional cell carcinomas of the renal pelvis, ureter and urethra [2]. The male:female incidence ratio is 2:1, though adjusted rates vary from 4.8–11.3 cases per 100,000 population in men, to 2.3–4.1 in women, depending upon the geographic area in question [2]. It has been argued that the growing incorporation in recent decades of new diagnostic techniques, such as echography, computed tomography, and magnetic resonance imaging, may have had an influence on the observed rise in incidence [5], though the most recent reviews conclude that the described increase is associated with the rise in prevalence of this tumor's risk factors [6].

Established risk factors for kidney cancer include cigarette smoking, obesity, diabetes, and hypertension [7]. However, these factors are thought to explain only half the incidence [5,8]. In the scientific literature other risk factors have been proposed, such as ingestion of some drugs (phenacetin, diuretics) [9], intake of certain dietary components (doneness of red meat) [10], or consumption of tea and coffee, with controversial results [7]. With respect to alcohol consumption, most studies (with differing designs) report no association with kidney cancer, though some do cite evidence of a possible protective effect found exclusively among women [11], exclusively among men [12], or in both sexes [13].

Another possible, less studied risk factor for kidney cancer is exposure to substances contained in drinking water, such as products resulting from disinfection of the water [14,15], nitrites, precursors of N-nitroso compounds [16,17] and some radionuclides. Regarding this last exposure, water from bedrock frequently contains higher concentrations of natural radionuclides than does water from other sources. Bladder and kidneys receive a radiation dose when radioactive isotopes are excreted into urine [18]. Studies conducted in Spain [19,20] and those undertaken in other countries [21] coincide in concluding that there is a wide variability of content in natural radionuclides depending upon the nature of the terrain and the geographic location, something that might in turn have a bearing on the spatial patterns of this disease.

Spatial analysis of health events (spatial epidemiology) is a discipline that, albeit still in the development phase, is already enjoying a space of its own in the field of health research [22,23]. Its ability to suggest and detect the possible sources of heterogeneity (generally of environmental origin) which determine the spatial patterns of incidence and mortality due to different diseases, imbues this tool with great interest in the sphere of epidemiology and public health. Its potential is, moreover, being reinforced by the ever increasing availability of geographically-indexed population mortality and incidence data, together with advances in computation techniques and Geographic Information Systems. These circumstances are favoring the analysis of the geographical distribution of health data with growing levels of disaggregation [24], a field that encompasses the so-called small-area studies.

The aim of this study was to show the spatial distribution patterns of kidney cancer in men and women, and discuss, in the light of known risk factors, possible hypotheses that would help explain these patterns and enhance existing knowledge about the large proportion of kidney tumors whose cause remains unknown.

## Methods

As case source, we used individual death entries for the period 1989–1998 corresponding to kidney cancer (International Classification of Diseases, 9^th ^Revision/ICD-9 code 189), broken down by town or city, nationwide. These data were furnished by the National Statistics Institute (*Instituto Nacional de Estadística *– *INE*) for the production of a municipal cancer mortality atlas, of which these results form part [25].

Municipal populations, broken down by age group (18 groups) and sex, were obtained from the 1991 census and 1996 municipal roll. These years correspond to the midpoints of the two quinquennia that comprise the study period (1989–1993 and 1994–1998). The person-years for each five-year period were obtained by multiplying these populations by 5.

Standardized mortality ratios (SMR) were calculated as the ratio of observed to expected deaths. To calculate expected cases, the overall Spanish mortality rates for the above two 5-year periods were multiplied by each town's person-years, broken down by age group, sex and quinquennium.

For map-plotting purposes, smoothed municipal relative risks (RRs) were calculated using the conditional autoregressive model proposed by Besag, York and Molliè (BYM). This model was introduced by Clayton and Kaldor [26], developed by Besag, York and Molliè [27], and subsequently applied in the field of ecological studies [28]. These models are based on fitting Poisson spatial models with observed cases as the dependent variable, expected cases as offset, and two types of random effects terms which take the following into account: a) municipal contiguity (spatial term); and b) municipal heterogeneity. The models were fitted using Markov chain Monte Carlo simulation methods with non-informative priors [29]. Posterior distributions of relative risk were obtained using WinBugs [30]. The criterion of contiguity used was adjacency of municipal boundaries. Convergence of the simulations was verified using the BOA (Bayesian Output Analysis) R program library [31]. Given the great number of parameters of the models, the convergence analysis was performed on a randomly selected sample of 10 towns and cities, taking 4 strata defined by municipal size. Convergence of the estimators was achieved before 100,000 iterations. For the maps shown, a "burn-in" (iterations discarded to ensure convergence) of 300,000 iterations was performed and the posterior distribution was derived with 5,000.

A Geographic Information System was used to plot municipal maps that depicted smoothed RR estimates and the distribution of the posterior probability (pp) that RR>1 (Bayesian version of p value). With regard to this indicator, we followed Richardson's criterion [32], which recommends that probabilities above 0.8 should be deemed significant. Separate analyses were performed for men and women.

## Results

From 1989 to 1998, a total of 14116 kidney cancer deaths were registered in Spain, 9431 among men and 4685 among women. In 5220 towns and cities no death due to this cause was registered. Using these data and conventional computers, it was possible to compile and ascertain the posterior distribution of relative risk on the basis of a single spatial model that included all of Spain's 8077 towns and cities and the 46398 adjacencies existing between them.

**Figure 1:**
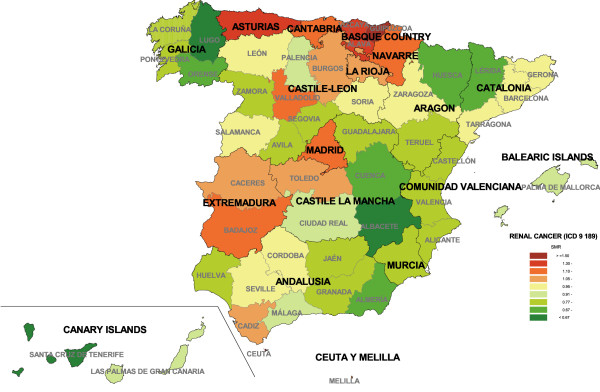
Provincial distribution of kidney cancer mortality: Spain, 1989–1998.

To give an overall picture, Figure 1 depicts kidney cancer mortality by province for both sexes. There were only six provinces with SMRs greater than 1.15, namely, Alava, Guipuzcoa, Biscay, Navarre, Asturias and Badajoz. The lowest SMRs were registered in Galicia, Aragon, the Valencian Region, Murcian Region and Mediterranean provinces of Andalusia. Table 1 shows the results for observed and expected deaths by province, in order to be able to assess the magnitude of the relative risk and differences by sex.

**Table 1:** Kidney cancer mortality by province: Spain, 1989–1998.

	TOTAL			MEN				WOMEN			
			
Province	obs	exp	SMR	obs	exp	SMR	p-val	obs	exp	SMR	p-val
ALMERIA	108	147.4	0.733	81	100.2	0.809	0.972	27	47.3	0.571	0.999
CADIZ	301	284.7	1.057	201	191.1	1.052	0.224	100	93.6	1.069	0.234
CORDOBA	266	265.6	1.002	183	176.7	1.036	0.301	83	88.9	0.933	0.713
GRANADA	236	262.2	0.900	144	177.3	0.812	0.994	92	84.9	1.084	0.203
HUELVA	131	147.9	0.886	87	98.2	0.886	0.861	44	49.7	0.885	0.768
JAEN	194	230.9	0.840	134	157.4	0.851	0.969	60	73.5	0.816	0.939
MALAGA	331	363.5	0.911	210	245	0.857	0.988	121	118.4	1.022	0.384
SEVILLE	506	483.9	1.046	330	319.5	1.033	0.268	176	164.4	1.071	0.172
											
HUESCA	80	107.1	0.747	49	75.1	0.652	0.999	31	32	0.970	0.520
TERUEL	66	79	0.835	39	54.8	0.712	0.984	27	24.2	1.114	0.248
ZARAGOZA	338	351.7	0.961	235	236.1	0.995	0.512	103	115.5	0.892	0.869
											
ASTURIAS	640	463.8	1.38	**447**	**304.8**	**1.466**	**< 0.001**	**193**	**158.9**	**1.214**	**0.004**
											
BALEARIC ISLES	240	263.1	0.912	169	177.1	0.954	0.713	71	86	0.825	0.945
											
LAS PALMAS	184	199.3	0.923	133	136.9	0.971	0.610	51	62.4	0.818	0.919
SANTA CRUZ	135	215.7	0.626	93	146.5	0.635	1	42	69.3	0.606	1
											
CANTABRIA	234	206.7	1.132	**163**	**136.5**	**1.194**	**0.012**	71	70.2	1.011	0.431
											
ALBACETE	85	132.3	0.642	54	91.2	0.592	1	31	41.1	0.755	0.937
CIUDAD REAL	174	189.9	0.916	104	127.6	0.815	0.982	70	62.3	1.124	0.149
CUENCA	72	103.1	0.698	43	71.4	0.602	1	29	31.7	0.916	0.640
GUADALAJARA	66	72.8	0.907	46	50.9	0.904	0.725	20	21.9	0.912	0.608
TOLEDO	223	209.7	1.063	150	143.6	1.044	0.281	73	66.1	1.104	0.180
											
AVILA	76	89.1	0.853	54	61.7	0.876	0.819	22	27.4	0.802	0.826
BURGOS	163	154.4	1.056	114	105.1	1.084	0.180	49	49.3	0.995	0.477
LEON	230	237.6	0.968	137	160.8	0.852	0.969	**93**	**76.8**	**1.210**	**0.032**
PALENCIA	76	82.2	0.924	55	54.8	1.003	0.454	21	27.4	0.767	0.872
SALAMANCA	165	166.4	0.992	100	112.3	0.891	0.867	**65**	**54.1**	**1.200**	**0.065**
SEGOVIA	56	71.9	0.779	39	49.1	0.795	0.918	17	22.8	0.747	0.868
SORIA	52	53.3	0.976	37	36.5	1.015	0.422	15	16.8	0.891	0.613
VALLADOLID	197	176	1.119	128	118.3	1.082	0.174	69	57.7	1.197	0.063
ZAMORA	93	114.5	0.812	60	77.8	0.771	0.978	33	36.7	0.898	0.697
BARCELONA	1710	1669	1.025	1125	1098.6	1.024	0.208	585	570.4	1.026	0.262
GERONA	211	204	1.034	140	138.9	1.008	0.440	71	65.1	1.091	0.212
LERIDA	125	163.5	0.765	83	113.8	0.729	0.998	42	49.7	0.845	0.847
TARRAGONA	212	219.5	0.966	145	150.1	0.966	0.641	67	69.4	0.965	0.583
											
ALICANTE	401	459.8	0.872	283	313.3	0.903	0.956	118	146.4	0.806	0.991
CASTELLON	164	181.8	0.902	110	124.1	0.886	0.890	54	57.8	0.935	0.659
VALENCIA	676	749.8	0.902	462	500.8	0.923	0.958	214	249.1	0.859	0.987
											
BADAJOZ	286	242.2	1.181	179	161.6	1.108	0.081	**107**	**80.6**	**1.327**	**0.002**
CACERES	181	169.6	1.067	110	114	0.965	0.622	**71**	**55.6**	**1.277**	**0.020**
											
CORUNNA	377	426.9	0.883	243	278.1	0.874	0.983	134	148.8	0.900	0.881
LUGO	132	207.4	0.637	80	141.3	0.566	1	52	66	0.787	0.956
ORENSE	130	191.1	0.68	75	127.6	0.588	1	55	63.5	0.866	0.842
PONTEVEDRA	274	311.6	0.879	179	199.9	0.895	0.927	95	111.7	0.851	0.940
											
MADRID	1788	1588.3	1.126	**1234**	**1035.2**	**1.192**	**< 0.001**	554	553.1	1.002	0.474
											
MURCIA	266	338.6	0.786	185	229	0.808	0.998	81	109.6	0.739	0.997
											
NAVARRE	249	209.8	1.187	170	138.8	1.225	0.005	79	71	1.112	0.157
											
ALAVA	123	91.4	1.345	**82**	**62.3**	**1.316**	**0.007**	**41**	**29.1**	**1.408**	**0.014**
GUIPUZCOA	360	238.5	1.509	**227**	**157.3**	**1.443**	**< 0.001**	**133**	**81.2**	**1.637**	**< 0.001**
VIZCAYA	563	406.6	1.385	**386**	**269.7**	**1.431**	**< 0.001**	**177**	**136.9**	**1.293**	**< 0.001**
											
LA RIOJA	121	111.2	1.088	87	75.6	1.151	0.088	34	35.6	0.955	0.563
											
CEUTA	32	17.3	1.847	**19**	**11.6**	**1.642**	**0.015**	**13**	**5.7**	**2.262**	**0.002**
MELILLA	17	13.8	1.23	8	9.1	0.875	0.564	**9**	**4.7**	**1.926**	**0.021**

Observed and expected cases, SMRs and p-values shown for both sexes, men and women. Provinces grouped by autonomous region.

SMR: Standardised mortality ratio

**Figure 2:**
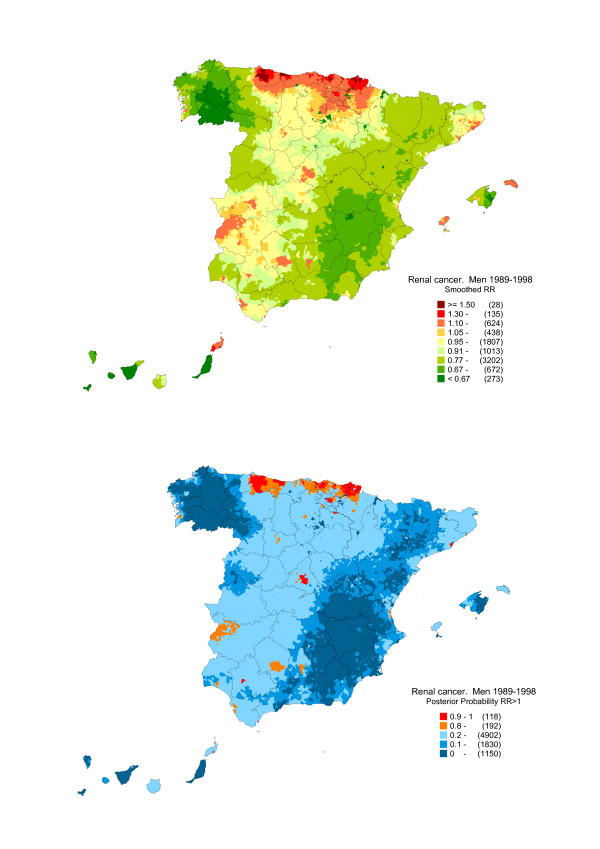
**Municipal distribution of kidney cancer mortality in men: Spain, 1989–1998.** Distribution pattern of the smoothed relative risk under the BYM model and posterior probability of RR being greater than 1.

**Figure 3:**
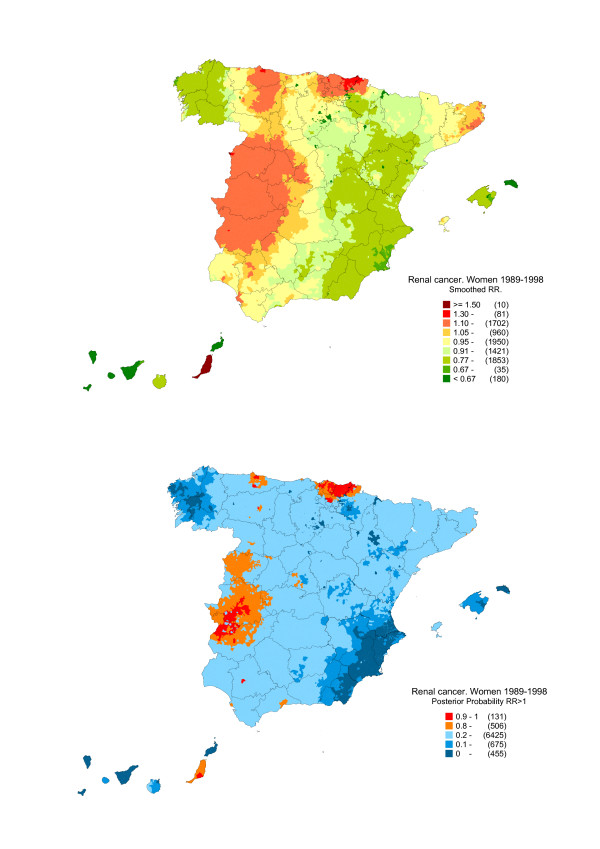
**Municipal distribution of kidney cancer mortality in women: Spain, 1989–1998.** Distribution pattern of the smoothed relative risk under the BYM model and posterior probability of RR being greater than 1.

Figures 2 and 3 depict the distribution of: a) the smoothed RRs for kidney cancer in men and women; and, b) the posterior probability (pp) that RR>1. This second map "filters" the previous one, flagging the areas in which excess mortality is more likely.

As the patterns for each sex displayed notable similarities and differences [25], the respective results are shown separately. The maps show that the highest mortality in both sexes was concentrated in towns along the Bay of Biscay coastline of the Asturian, Cantabrian and Basque Country Autonomous Regions. The different mortality pattern registered by women was noteworthy, with excess risk in the west of the country, covering wide swathes of Salamanca and Extremadura. In men, the only excess mortality observed in this area exclusively affected two towns, i.e., Badajoz and Mérida.

## Discussion

Municipal maps of kidney cancer distribution display a marked geographic pattern, with excess mortality in both sexes chiefly in towns along the Bay of Biscay, including areas of Asturias, the Basque Country and, to a lesser extent, Cantabria. Among women, special mention must be made of the existence of a strikingly singular pattern, not in evidence for any other tumors and marked by excess risk in towns situated in the Salamanca area and Extremaduran Autonomous Region. This difference would lead one to postulate the existence of different exposures of environmental origin in the various regions.

Possible misclassification errors involved in the study of RCC mortality render a high degree of caution necessary when it comes to assessing the patterns observed in the results. Despite the fact that undercertification of renal cancers has been reported by studies on the accuracy of death certification in Spain [33], there are not too many arguments that would support possible inconsistencies and differences of criteria in the coding of death certificates, i.e., if the certification/coding of deaths were not correct, the errors would not necessarily follow any pattern, and there would be no agreement between incidence and mortality data. Another explanations for possible differences in kidney cancer mortality across the country, as differences in survival rates due to the distribution of tumor stage at diagnosis are difficult to maintain because the universal accessibility to the health care system. Bearing in mind the characteristics of the Spanish National Health Care System, we would have no reason to suspect that there might be differential access to health care and diagnosis between regions.

On examining the incidence data reported in officially certified Spanish population cancer registers and published in "Cancer Incidence in Five Continents, Volume IX" [2], the coincidence with mortality patterns can be appreciated. The highest incidence rates for both sexes were those registered in the Basque Country and Asturian Autonomous Communities (1998–2001), there being no data for the Provinces of Salamanca, Cáceres and Badajoz, as population-registry-based information is still lacking in these areas.

Smoking habit is the most widely established risk factor for both histologic types of kidney cancer [34]. Yet, the patterns shown in the maps presented here diverge considerably from the patterns described for lung and bladder cancer among men and women, respectively [35]. Neither is there any concordance between the geographic pattern observed and the prevalence of obesity. Specifically, insofar as prevalence of obesity among women aged 35–64 years is concerned, only one of the provinces with excess risk, Badajoz, figures among the 10 provinces with highest prevalences of obesity [36].

Areas with excess kidney cancer mortality along the coast of the Bay of Biscay also register higher mortality due to other smoking-related tumors, among men in particular. As has been remarked above, however, the pattern of kidney cancer mortality is not concordant with that observed for other causes of death strongly related with tobacco [25]. Similarly, excess mortality due to ischemic heart or cerebrovascular diseases is not observed along the coast of the Bay of Biscay, so that other risk factors described (cigarette smoking, obesity, diabetes, and hypertension)[7] would not appear to explain this pattern. Attention should be drawn to the fact that excess kidney cancer on the Bay of Biscay is located in a heavily industrialized area (mining and heavy metal industry). As shown in the European Pollutant Emission Register (EPER), this area receives the highest industrial cadmium, arsenic, nickel and benzene emissions in Spain [37], substances which are classified in IARC group 1 in terms of their carcinogenic activity (human carcinogens) [38] and for which an association with renal cancer has been documented [39-43]. Nonetheless, no references could be found in the literature which analyze the possible influence of industrial pollution on RCC.

The pattern displayed by female mortality in western parts of central mainland Spain (Salamanca, Cáceres, and Badajoz) is somewhat fuzzy, with smoothed RRs not exceeding 1.3, and posterior probabilities of RR>1 exceeding 0.80 over wide areas and reaching 0.90 in very few towns, something that could be interpreted as a consequence of the smoothing procedure. However, this possible artifact should affect other areas, and yet it would not appear to do so.

Taking the late introduction of the smoking habit among Spanish women into account [36], the peculiarities of the pattern displayed in women can be assumed to be attributable to other factors (dietary or environmental). Reviewing the known risk factors and possible explanations for the higher risk of this disease among women in the province of Salamanca and Extremadura, there are very few environmental components: 1) to which women might be more exposed; and 2) for which the kidney might be the target organ. Given the geologic composition of the terrain in these provinces [44,45], consumption of drinking water is one of the possibilities that could fit such a scenario. As men consume more alcohol than women, their liquid intake can be surmised to involve a lower consumption of publicly supplied water, water that may contain components which, in the context of chronic exposure, could well cause some form of renal toxicity. It has been shown that chronic ingestion of certain radionuclides (uranium) in drinking water may affect renal function [46]. Due to the lower prevalence of smokers among women, the effect of environmental factors might be more visible among them, since the presence of a risk factor such as tobacco, with a far higher RR, would partially mask the influence of environmental factors whose effect magnitude was substantially lower.

Salamanca, Cáceres and Badajoz are all provinces rich in uranium ore, and in one of them concentrations of radionuclides (U, Th, Ra) in groundwater (bedrock) have been found which were 5–30 times higher than in surface water, whereas one third of the samples contained Ra concentrations that exceeded the recommended limits [45]. In this respect, it must be said that the percentage of the population relying on a groundwater supply is 23% in Castile-León and 33% in Extremadura [47].

On the other hand, there is evidence of the existence of other granitic areas in Galicia, where natural radiation is high [44] and yet no excess risk of kidney cancer has been observed. The radiologic situation of this area's drinking water is not known because no systematic studies have been undertaken [48]. In this Autonomous Region the percentage of the population supplied with groundwater is 12%, a figure very much lower than that for Spain as a whole [47]. Due to their chalky soils and highly mineralized waters, the areas in Spain that register the maximum consumption of bottled water are the Balearic Isles, Catalonia, the Valencian Region, Castile-La Mancha, Murcia, and Aragon [49], and these same areas are shown in the maps as having lower-than-expected kidney cancer mortality.

A growing number of cohort studies have reported that alcohol consumption has a protective effect as against risk of RCC: in men [50]; in women [12,51]; or in both [52]. The mechanism of action whereby alcohol serves to reduce RCC risk has not been elucidated. Some authors are of the opinion that it may be the ethanol itself that produces the protective effect, since the effect is not confined to any one type of alcoholic beverage. In the studies consulted, no estimate is made of the effect of the reduction in daily water intake attributable to consumption of alcoholic beverages. Mean daily intake of water in Spain is 1,574.4 ml (+- 327) among adults (aged over 17 years) [53]. Regardless of the fact that moderate consumption of alcohol has a slightly protective effect vis-à-vis RCC [54], persons who received an important part of their liquid intake in the form of alcoholic beverages (e.g., wine or beer) would be far less exposed to agents conveyed in publicly piped drinking water, whether from surface or underground sources.

The nationwide survey on diet and eating habits points to estimated alcohol consumption as being very much higher in men. The prevalence of male alcohol consumers in these provinces is extremely high (72% and 74% male vs. 38% and 47% female alcohol consumers in Extremadura and Castile-Leon, respectively) [36].

Exposure to products resulting from the disinfection of water is also being targeted for study in connection with urinary bladder tumors [55,56], though ecologic mortality [14] and experimental animal research studies suggest a possible relationship with renal tumors [57,58].

Other compounds related with the water supply are nitrates. Nitrate has steadily accumulated in our water supply and is the most common chemical contaminant in the world's groundwater aquifers [17]. In agricultural regions, nitrate inputs are largely due to nitrogen fertilizer use [59]. Nitrates are a precursor in the formation of N-nitroso compounds (NOC), most of which are animal carcinogens [60]. Specific NOC cause renal cancers in animal studies [61]. Other sources of NOC exposure include preformed NOC found in preserved meats and fish, tobacco, and certain occupational exposures [62].

Nitrate contamination of groundwater in Spain seriously affects (> 50 mg/l) the entire Mediterranean seaboard, which displays no excess risks of kidney cancer in either sex. Among the most affected inland areas are the Manchegan plain, the Ebro delta and some sections of the Guadalquivir valley. Locally, the presence of nitrates affects different areas of the Duero (central Duero, Esla-Valderaduey, and Arenales), Tagus (La Alcarria, Tiétar and Ocaña), Sur (Campo de Níjar, Dalías, and Fuente Piedra), and Segura river basins (Campo de Cartagena, Guadalentín, and Vegas del Segura). At a lower degree of intensity (25 through 50 mg/l), this contamination affects many water supply points across Asturias and the Basque Country [63].

## Conclusion

Municipal kidney cancer distribution maps display a marked geographic pattern. The maps show that highest mortality in both sexes is concentrated in towns along the Bay of Biscay, covering areas of Asturias, the Basque Autonomous Region and, to a lesser extent, Cantabria. The reasons for this pattern of distribution are not at all clear, and it would thus be of interest to study the effect of industrial emissions and immissions on this disease. Interventions targeted at decreasing prevalence of smoking, obesity and, perhaps, hypertension might tend to stabilize or reduce the incidence and mortality, and only go some way towards mitigating the geographic differences displayed. The different pattern registered by female mortality in towns in the Salamanca area and the Extremaduran Autonomous Region is noteworthy but, subject in all cases to the necessary caution, this could in part be explained by exposures linked to the geologic composition of the terrain and, in turn, to the local drinking water.

## Competing interests

The authors declare that they have no competing interests.

## Authors' contributions

GLA, MP, NA, and BPG were all involved in designing the study. GLA and RR performed the statistical analysis. GLA wrote the first draft of the manuscript to which all authors subsequently contributed. All authors made contributions to the statistical analyses and interpretation of results, and revised the manuscript for important intellectual content. All authors read and approved the final manuscript.

## Pre-publication history

The pre-publication history for this paper can be accessed here:


